# A randomised, double- blind, cross-over study investigating the prebiotic effect of agave fructans in healthy human subjects

**DOI:** 10.1017/jns.2014.68

**Published:** 2015-03-13

**Authors:** P. Ramnani, A. Costabile, A. G. R. Bustillo, G. R. Gibson

**Affiliations:** 1 Department of Food and Nutritional Sciences, University of Reading, Reading RG6 6AP, UK; 2 Bustar Alimentos, SAPI de CV, Efrain Gonzalez Luna 2503, Col Arcos Sur, 44130 Guadalajara, Jalisco, Mexico

**Keywords:** Agave fructans, Gut microbiota, Prebiotics, DGGE, denaturing gradient gel electrophoresis, FISH, fluorescent *in situ* hybridisation, sIgA, secretory IgA, SS1, steady state 1, SS2, steady state 2

## Abstract

This placebo-controlled, randomised, double-blind, cross-over human feeding study aimed to determine the prebiotic effect of agave fructans. A total of thirty-eight volunteers completed this trial. The treatment consisted of 3 weeks' supplementation with 5 g/d of prebiotic agave fructan (Predilife) or equivalent placebo (maltodextrin), followed by a 2-week washout period following which subjects were crossed over to alternate the treatment arm for 3 weeks followed by a 2-week washout. Faecal samples were collected at baseline, on the last day of treatment (days 22 and 58) and washout (days 36 and 72), respectively. Changes in faecal bacterial populations, SCFA and secretory IgA were assessed using fluorescent *in situ* hybridisation, GC and ELISA, respectively. Bowel movements, stool consistencies, abdominal comfort and mood changes were evaluated by a recorded daily questionnaire. In parallel, the effect of agave fructans on different regions of the colon using a three-stage continuous culture simulator was studied. Predilife significantly increased faecal bifidobacteria (log_10_ 9·6 (sd 0·4)) and lactobacilli (log_10_ 7·7 (sd 0·8)) compared with placebo (log_10_ 9·2 (sd 0·4); *P* = 0·00) (log_10_ 7·4 (sd 0·7); *P* = 0·000), respectively. No change was observed for other bacterial groups tested, SCFA, secretory IgA, and PGE_2_ concentrations between the treatment and placebo. Denaturing gradient gel electrophoresis analysis indicated that bacterial communities were randomly dispersed and no significant differences were observed between Predilife and placebo treatments. The *in vitro* models showed similar increases in bifidobacterial and lactobacilli populations to that observed with the *in vivo* trial. To conclude, agave fructans are well tolerated in healthy human subjects and increased bifidobacteria and lactobacilli numbers *in vitro* and *in vivo* but did not influence other products of fermentation.

The importance of human gastrointestinal microbiota is becoming increasingly recognised. Diet–microbe interactions within the colon can result in a number of health benefits: protection from invading pathogens, modulation of immune system, production of vitamins and removal of carcinogens^(^^1^^–^^3^^)^. Selectively modulating the gut microbial activities is the basis of the prebiotic concept that advocates targeting beneficial bacteria through non-viable food ingredients^(^^4^^,^^5^^)^.

To date most attention has been focused on the prebiotic potential of fructo-oligosaccharides and *trans*-galacto-oligosaccharides. However, other fibres including resistant starches or dextrins, glucans, gums and pectins are also increasingly being recognised as having prebiotic potential^(^^6^^,^^7^^)^.

Fructans have been classified according to their structure and fructosyl linkage as inulin, levan, graminans, neoseries levan and neoseries graminans^(^^8^^)^. The importance of inulin-type fructans with linear β (2 → 1) linkages in human and bowel health is well established both *in vitro* and *in vivo*^(^^3^^,^^9^^)^. They have been consistently associated with increases in populations of bifidobacteria and lactobacilli and production of desirable fermentation endproducts^(^^3^^,^^10^^,^^11^^)^. The rate and extent of fermentation of fructans is influenced by the degree of polymerisation^(^^12^^–^^14^^)^. Several studies have investigated linear-chain fructans^(^^3^^)^; however, there are few data available on branched-chain fructans^(^^15^^)^.

Agave plants have been historically known to be an important source of natural fibre and alcoholic beverages in Mexico^(^^8^^)^. Fructans from agave demonstrate wide diversity with a complex and highly branched mixture of fructo-oligosaccharides and fructans containing both β (2 → 1) and β (2 → 6) linkages with internal (neoseries fructans) and external (graminans fructans) glucose units^(^^16^^)^. Different agave species show variation in proportion of glucose polymerisations and β (2 → 1) and β (2 → 6) linkages^(^^17^^)^. Structural branching of agave fructans may result in altered gut bacterial modulation and fermentation profile compared with inulin-type fructans. Scanty information on the prebiotic properties of agave fructans justifies the present study where fructans from *Agave tequilana* Weber var. *azul.* (Predilife) were tested in an *in vitro* gut model and human trial. A randomised, placebo-controlled, cross-over design was chosen due to its robustness and reduced intra-individual variability.

Fermentation of prebiotics is primarily anaerobic and thus leads to the production of SCFA. The structure of carbohydrates and gut microflora influence SCFA profiles^(^^7^^,^^14^^)^. Therefore, in addition to studying the effect of agave fructans on the composition of gut microbiota, SCFA concentrations were also measured.

The prebiotic modulation of bacterial communities has been shown to influence the host immune functions, including up-regulation of immunoglobulins and reduction in prostaglandins^(^^18^^,^^19^^)^. Consequently, the present study determined levels of the two biomarkers: secretory IgA (sIgA) and PGE_2_.

## Materials and methods

### Test products

The prebiotic was provided to volunteers as sachets containing 5 g Predilife, a purified powder extracted from *Agave tequilana* Weber var. *azul.* The preparation of Predilife has been described previously^(^^15^^)^. The placebo was a sachet containing 5 g of commercially available Maltodextrin Star DRI® 10 with Dextrose Equivalent 10 (Tate and Lyle). All test and placebo products were packaged and blinded by Bustar Alimentos, Mexico. The products were only distinguishable by the colour of the label. The nutritional information of the products is given in Table 1.

**Table 1.** Nutritional information of placebo and Predilife

Nutrients (per 5 g dose)	Placebo*	Predilife†
Energy		
kJ	66·32	34·12
kcal	15·6	8·15
Protein (g)	0	Trace
Carbohydrates (g)	3·90	4·90
Sugars (g)	0·78	0·20
Fat (g)	0	Trace
SFA (g)	0	0
Cholesterol (mg)	0	0
Dietary fibre (g)	0	4·70
Of which fructans (inulin) (g)	0	4·70
Na (mg)	8·59	2·50

* Placebo ingredients: Maltodextrin Star DRI 10, orange flavour, orange colour, sucralose.

† Predilife ingredients: Predilife agave fructans, orange flavour, orange colour, sucralose.

### Study design

**Fig. 1.**
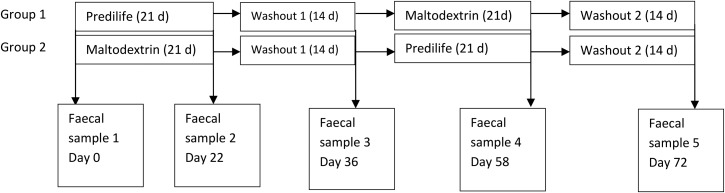
Study design.

The dietary intervention study was a double-blind, randomised, placebo-controlled cross-over trial. At 14 d before the start of the study, volunteers were asked to follow a restricted diet; no consumption of pre- or probiotics was allowed. A total of thirty-eight subjects were randomly allocated into one of the two groups using an automated randomisation sequence generated by a web-based tool (randomisation.com). The first group (*n* 19) consumed Predilife (5 g/d) for 3 weeks followed by a washout period for 2 weeks; they then consumed an equivalent placebo (5 g/d) for 3 weeks followed by a 2-week washout period. The second group (*n* 19) received a placebo for the first 3 weeks followed by a 2-week washout period, Predilife for 3-weeks followed by a 2-week washout (Fig. 1). No trial product was consumed during the 2-week washout period. During the intervention period, study subjects were instructed to consume one sachet of the provided treatment by mixing the test powder in 300 ml of water with breakfast every day. During the trial, volunteers and researchers were unaware which product was taken by which participant.

### Subjects

A total of forty healthy human volunteers were recruited from the Reading area of the UK to participate in the present study. The subjects were recruited by advertising on notice boards and emails were sent to all staff at Reading University. In addition, letters were sent to volunteers registered with the Sensory Dimensions (Reading, UK) database. Two volunteers were prescribed antibiotics and were thus excluded from the study, before the first and the second feeding period, respectively. For analysis purposes, thirty-eight volunteers, nineteen males and nineteen females were included. Their mean age was 35 (sd 8·0) years and average BMI was 24·1 (sd 3·0) kg/m^2^. Written informed consent was obtained from all subjects. The study was reviewed and approved by the University of Reading Ethics Committee. The study protocol was conducted according to guidelines in the Declaration of Helsinki.

### Inclusion and exclusion criteria

#### Inclusion criteria

Inclusion criteria were a signed consent form, aged 18–50 years inclusive, BMI 18–30 kg/m^2^ inclusive, non-smoking and good general health.

#### Exclusion criteria

Volunteers were excluded from the trial if there was evidence of physical or mental disease or major surgery. Volunteers with a history of drug or alcohol abuse, severe allergy, abnormal drug reaction, pregnant, lactating or planning pregnancy were excluded from the study. Intake of an experimental drug within 4 weeks before the study, former participation in a probiotics, prebiotics or laxative trial within the previous 3 months, use of antibiotics within the previous 6 months, chronic constipation or other chronic gastrointestinal complaints (for example, irritable bowel syndrome) were all exclusion criteria.

### Diet and medication requirements during the trial

Intake of prebiotics, probiotics and drugs active on gastrointestinal motility, antibiotic treatment or any class of laxatives were not permitted. Subjects were not allowed to participate in any other nutritional or pharmaceutical trials during the study. Any medication taken was recorded in volunteer diaries. Volunteers were instructed not to alter their usual diet or fluid intake during the study.

### Volunteer diaries

Volunteers were asked to keep diaries throughout the study to record stool frequency and consistency, abdominal pain, intestinal bloating and flatulence on a daily basis. Energetic status, happiness, alertness and stress levels as compared with normal were also recorded. Any concomitant medication, adverse events and failure to consume any treatments were also recorded by the volunteers.

In addition, volunteers were asked to record the time of consumption of the product for measuring compliance. Volunteers were instructed to return any unused sachets at the end of the intervention. They were considered compliant for the product if they consumed at least 90 % of the product over the 3-week intervention. They were also asked not to alter their usual diet and fluid intake and record changes, if any.

### Stool sample collection and processing

Freshly voided stool samples collected in plastic pots were stored in an anaerobic cabinet (10 % H_2_, 10 % CO_2_, 80 % N_2_; Don Whitley Scientific) at 37°C and processed within 2 h of voiding. Faecal samples were collected before and after treatment with either agave fructan or maltodextrin and washout on days 0, 22, 36, 58 and 72 (Fig. 1). Samples were diluted 1:10 (w/w) in PBS (0·1 m, pH 7·0) and homogenised in a stomacher (Seward) at normal speed for 2 min. The faecal slurry was transferred to 50 ml sterile plastic centrifuge tubes containing 2 g glass beads (diameter 5 mm) and vortexed for 30 s. Samples were then centrifuged at 400 ***g*** for 5 min at room temperature and supernatant fractions processed for fluorescent *in situ* hybridisation (FISH) and SCFA analysis. The pellet was resuspended in 500 µl of autoclaved PBS–glycerol (1:1) for DNA extraction. A quantity of 1 g of faeces was collected in microcentrifuge tubes and stored at –20°C for ELISA.

### Fluorescent *in situ* hybridisation

Synthetic oligonucleotide probes targeting specific regions of the 16S rRNA labelled with fluorescent Cy 3 were utilised for enumeration of bacterial populations. The faecal supernatant fractions obtained as described above were fixed in 4 % (w/v) paraformaldehyde and hybridised with appropriate probes as described by Vulevic *et al.*^(^^18^^)^. The probes used were Bif164^(^^20^^)^, Lab158^(^^21^^)^, Erec482^(^^22^^)^, Ato291^(^^23^^)^, Bac303^(^^24^^)^, Rrec584^(^^22^^)^, Eco1531^(^^25^^)^ and Eub338 mix^(^^26^^)^ specific for bifidobacteria, *Lactobacillus*/*Enterococcus* spp., *Eubacterium*/*Clostridium coccoides* group, *Atopobium* spp., *Bacteroides*, *Roseburia*/*Eubacterium rectale* spp., *Escherichia coli* and total bacteria, respectively. Slides were examined under an epifluorescence microscope (Eclipse 400; Nikon) using the Fluor 100 lens. At least fifteen random fields of view were counted for each well and microbial counts expressed as log _10_ bacterial cells per g dry weight faeces.

### SCFA analyses

Fermentation output was determined by measuring changes in faecal SCFA concentrations in the collected supernatant fractions. Acetate, propionate, butyrate, isobutyrate, valerate, isovalerate and caproate were analysed in the collected samples as their salyl derivatives by GC^(^^27^^)^. The organic acids were extracted by addition of 0·5 ml concentrated hydrochloric acid and 2 ml diethyl ether. *N*-tert-butyldimethylsilyl-*N*-methyltrifluoroacetamide (MTBSTFA) was used to derivatise the samples at 80°C for 20 min. Samples were then run through a 5890 series II GC system (Hewlett Packard) fitted with a SGE-HT5 (0·32 mm × 25 m × 0·1 µm) (J & W Scientific) and a flame ionisation detector. Injector, oven and detector were set at 275, 250 and 275°C, respectively. A quantity of 1 µl of each sample was injected with a run time of 10 min. Peaks were integrated using the Atlas Lab managing software (Thermo Lab Systems). Fatty acid concentrations were calculated in mmol/l by comparing their peak areas with standards.

### Immunological analysis

Faecal sIgA (Oxford Biosystems) and PGE_2_ (Neogen Corp.) were measured by ELISA using commercially available kits and instructions provided by the manufacturers. Absorption was measured using an ELISA reader (GENios; Tecan). A calibration curve was constructed using a range of standards which were then used to assay the immunological marker in each of the samples. The results were expressed as µg/g or pg/g faeces (wet weight).

### Bacterial DNA extraction and PCR–denaturing gradient gel electrophoresis

Bacterial cell pellets collected after centrifugation and resuspension in PBS–glycerol, frozen at –20°C, were thawed on ice. Bacterial DNA was extracted using the FastDNA Spin kit (Qbiogene) according to the manufacturer's instructions. DNA was resuspended in 50 µl of sterile water, quantified using a NanoDrop ND-1000 spectrophotometer (NanoDrop Technologies) and stored at –20°C. PCR using universal P2 and P3 primers was performed as previously reported^(^^28^^)^. Approximately 5 µl of each PCR product were applied to denaturing gradient gel electrophoresis (DGGE) using a VWR CTV400-DGGE unit (VWR International). The polyacrylamide gel (acrylamide-bisacrylamide, 37·5:1; Bio-Rad) had a linear denaturing gradient of 30–70 %. Electrophoresis was run in 0·5× Tris acetate EDTA (TAE) buffer (made from 50× concentrate; Fischer) at 100 V and 60°C for 16 h. Gels were silver stained and scanned using a Cannon scanner (Lide 50; Cannon) and analysed using FPQuest Software version 4·5 (Bio-Rad). In order to compensate for gel-to-gel differences and distortion due to electrophoresis, DGGE patterns were aligned and normalised using a reference ladder composed of baseline sample from one volunteer. After normalisation, bands from each sample were defined using appropriate densitometric curves. Bands constituting less than 1 % of the total band area were omitted from further analysis. Similarity between DGGE profiles was calculated using the Pearson correlation. Clustering of profiles was done using the unweighted pair-group method using arithmetic average.

### Three-stage continuous culture model

Parallel to the human study, *in vitro* testing was carried out using a three-stage continuous culture model of the human colon^(^^29^^)^. The model consisted of three glass vessels with increasing working volume aligned in series. The first vessel simulated the proximal colon and had an operating volume of 280 ml and was fed with the growth medium. The second vessel, simulating the transverse colon, had an operating volume of 300 ml and was fed from the overflow of the first vessel. The third vessel simulating the distal colon had an operating volume of 320 ml and was fed from the overflow of the second vessel. Culture fluid from the third vessel was vented into a waste container. All vessels were continuously stirred and maintained at 37°C by a circulating water jacket. The pH of the vessels was maintained at 5·4, 6·2 and 6·7 for vessels 1, 2 and 3, respectively, by using pH controllers (Electrolab) pumping in 0·5 m-HCl/NaOH solutions as required. The system was kept anaerobic by continuously sparging with O_2_-free N_2_ through the liquid (about 15 ml/min) in all vessels. The culture medium consisted of the following components (g/l): starch 5; peptone water 5; yeast extract 4·5; tryptone 5; NaCl 4·5; KCl 4·5; mucin 4; casein 3; pectin 2; xylan 2; arabinogalactan 2; NaHCO_3_ 1·5; MgSO_4_.7H_2_O 1·25; guar gum 1; inulin 1; cysteine HCl 0·8; KH_2_PO_4_ 0·5; K_2_HPO_4_ 0·5; bile salts no. 3 0·4; CaCl_2_.6H_2_O 0·15; haemin 0·05; vitamin K 0·01; FeSO_4_.7H_2_O 0·005; Tween 80 1 ml. A quantity of 4 ml rezarsurin was added at a concentration of 0·025 % (w/v) to the medium as an indicator of anaerobicity.

Faecal inoculum was collected from one healthy human volunteer, aged 22 years, who had not taken antibiotics 6 months before sample collection. The sample was diluted 1:5 (w/v) in PBS (0·1 m; pH 7·4) and homogenised in a stomacher (Seward) for 2 min. Each vessel was inoculated with 100 ml fresh faecal slurry. After 24 h of inoculation, the medium flow was initiated and the system ran for eight full volume turnovers (16 d) to allow steady state 1 (SS1) to be achieved. At SS1, 5 ml samples were collected from each vessel for three consecutive days and centrifuged at 13 000 ***g*** for 10 min. The supernatant fractions were fixed in 4 % (w/v) paraformaldehyde for FISH analysis. For SCFA analysis the supernatant fractions were stored at –20°C and analysed by GC. Total bacterial counts and SCFA concentrations were analysed over three consecutive days to confirm SS1. Three gut models were run using faecal samples from the same donor, namely Synergy 1 (a prebiotic – fructo-oligosaccharide inulin mix from Orafti), Predilife and maltodextrin. After SS1, each of the test products was added to the first vessel in 5 g doses each day until steady state 2 (SS2) was reached after further eight volume turnovers (33 d). Samples were obtained as above for measuring any changes in gut microbiota using FISH and SCFA analysis for three consecutive days until a second steady state (SS2) was established.

### Statistical analyses

A total of forty healthy human volunteers were recruited based on statistical power calculation. The sample size was determined to detect a 0·5 log_10_ change in bifidobacterial counts with power set at 0·9, and a significance level of 0·05 based on our previous prebiotic studies in human volunteers conducted with the same microbiological techniques^(^^30^^,^^31^^)^.

Statistical analysis was performed on bacterial counts (log_10_ cells/g faeces) and fermentation characteristics using SPSS software (version 19; SPSS Inc.). Data from volunteers that completed the intervention were included in the analysis. Statistical significance of the overall treatment effect was judged using linear mixed models with compound symmetry repeated covariance format. Treatment, period and sequence were fixed effects, period was a repeated measure and participant was a random effect. All models were adjusted for age, sex, BMI, baseline values, sequence and period. Treatment, sequence and period terms were used to test for the presence of a carryover effect. In exploratory terms, there were no significant period order effects noted. The effects of age, sex or BMI on treatment were assessed in each model by inserting an interaction between treatment and each of the terms one at a time. Stratified analyses were performed if either of the interactions was significant. Student's *t* tests were used to compare the pre-treatment and post-treatment period measurements within each treatment, bowel habits, gastrointestinal symptoms and mood data. Data for DGGE were analysed by using FPQuest Software version 4·5 (Bio-Rad). For all analyses, *P* < 0·05 indicated statistical significance.

## Results

### Subject characteristics and compliance

In total, forty volunteers entered the cross-over study (twenty female, twenty males); of these, two were excluded due to antibiotic intake. Therefore, a total of thirty-eight volunteers (nineteen female, nineteen males) aged 20–49 years (average age 35 (sd 8·0) years) with average BMI 24·1 (sd 3·0) kg/m^2^ completed the human trial. Compliance for product intake, as assessed by diary data of regular consumption of sachets and returned unused sachets, was good (95–100 %). None of the volunteers indicated alterations in diet or fluid intake and were thus complaint.

### Medication and adverse events

Volunteers had consumed a variety of over-the-counter drugs such as cold and flu remedies, anti-allergy tablets and painkillers. No extremes were observed and the level of medication was judged as representative of a typical UK population.

Among adverse events recorded in volunteer diaries, headache, cough and colds, fever, backache, toothache were recorded over the two treatment and washout periods. No serious adverse events were recorded.

### Faecal microbiota

Changes in bacterial numbers are shown in Table 2. Consumption of Predilife increased bifidobacterial numbers (log_10_ 9·6 (sd 0·4)) compared with placebo (log_10_ 9·2 (sd 0·4)). Levels of bifidobacteria returned to approximate baseline levels (log_10_ 9·2 (sd 0·2)) (*P* < 0·001) 2 weeks after intervention was stopped.

**Table 2.** Faecal bacterial numbers (log_10_ cells/g faeces) determined in thirty-eight volunteers by fluorescence *in situ* hybridisation in the placebo-controlled, double-blind, cross-over human feeding study investigating the effects of Predilife (5 g/d) as compared with the placebo maltodextrin (5 g/d) (Mean values and standard deviations)

	Predilife				Maltodextrin			
	Baseline		Treatment		Baseline		Treatment	
Bacterial group	Mean	sd	Mean	sd	Mean	sd	Mean	sd
Total bacteria	10·8	0·2	10·8	0·2	10·8	0·2	10·8	0·2
*Atopobium*	9·3	0·4	9·3	0·4	9·3	0·3	9·4	0·4
*Bacteroides*	9·9	0·1	9·9	0·1	9·9	0·1	9·9	0·1
*Bifidobacterium* spp.	9·2	0·4	9·6***††	0·4	9·2	0·3	9·2	0·4
*Eubacterium rectale*/*Clostridium coccoides*	10·1	0·2	10·2	0·3	10·1	0·2	10·1	0·4
*Clostridium histolyticum* group	7·8	0·3	7·9	0·3	7·9	0·3	7·9	0·3
*Lactobacillus*/*Enterococcus* spp.	7·3	0·6	7·7***†††	0·8	7·3	0·6	7·4	0·6
*Escherichia coli*	7·5	0·3	7·4	0·3	7·5	0·3	7·4	0·3
*Roseburia*/*Eubacteria* group	9·7	0·2	9·7	0·2	9·7	0·2	9·7	0·2

*** Mean value was significantly different from that for maltodextrin (*P* < 0·001).

Mean value was significantly different from that at baseline: †† *P* < 0·01, ††† *P* < 0·001.

Lactobacilli/enterococci numbers also significantly increased following Predilife treatment (log_10_ 7·7 (sd 0·8)) as compared with placebo (log_10_ 7·6 (sd 0·6)) (Table 2).

For all the other groups enumerated, no significant differences were observed.

### Analysis of bowel habits, intestinal comfort and mood

Table 3 summarises data on bowel habits, intestinal comfort and mood. No significant differences were recorded in the mean daily stool frequencies and consistencies with either treatment. However, some volunteers did report a borderline significant trend for more formed stools and decrease in constipation after consumption of Predilife (*P* = 0·08). No significant change in abdominal pain was observed. Predilife consumption led to increased intestinal bloating (mild to moderate) compared with maltodextrin (*P* < 0·05). No significant increase in flatulence was observed after consumption of Predilife compared with placebo. In addition, no significant differences in mood scores were observed with either treatment.

**Table 3.** Summary of bowel habit, intestinal comfort and general mood data recorded on a daily basis in volunteer diaries throughout the study‡ (Mean values and standard deviations)

	Predilife				Maltodextrin			
	Baseline		Treatment		Baseline		Treatment	
	Mean	sd	Mean	sd	Mean	sd	Mean	sd
Stool frequency (*n*/d)	1·2	0·4	1·4	0·5	1·1	0·3	1·3	0·4
Stool consistency								
Hard	6·9	15·2	6·6	11·6	7·3	11·4	7·9	17·1
Formed	73·4	22·4	55·0	34·9	74·1	25·1	71·3	27·0
Soft	20·1	25·1	38·5	36·4	16·9	23·2	20·5	22·1
Abdominal pain								
None	81·2	20·4	84·5	26·7	80·1	15·3	89·5	17·8
Mild	15·2	18·1	12·4	22·7	16·2	8·3	7·2	9·9
Moderate	2·1	15·2	2·5	11·7	2·8	4·1	1·8	5·7
Severe	0·2	1·3	0·6	3·9	0·9	2·1	1·4	8·5
Intestinal bloating								
None	85·2	18·2	79·7*†	29·0	84·1	25·1	84·2	27·7
Mild	10·1	11·8	14·4*†	18·9	11·2	20·3	9·9	16·1
Moderate	2·7	10·1	5·6*	19·4	2·5	7·8	4·8	13·9
Severe	0·3	0	0·3	1·5	0	0	1·1	4·9
Flatulence								
None	68·8	32·6	51·2	38·9	66·5	30·1	63·2	37·5
Mild	28·2	31·0	31·7	27·5	28·7	17·1	26·7	26·2
Moderate	5·9	8·9	13·7	21·3	4·7	8·2	8·4	14·8
Severe	0·8	2·3	3·6	9·8	0·3	5·7	1·6	5·9
Mood changes								
Happy								
Less than normal	8·1	18·1	5·1	8·8	4·2	18·1	4·2	9·9
Normal	88·2	12·2	89·2	17·0	90·1	15·2	93·1	13·7
More than normal	2·0	9·4	6·8	15·4	6·4	10·2	2·7	6·5
Alert								
Less than normal	4·5	9·1	3·6	9·6	3·0	8·0	2·9	6·6
Normal	95·2	19·2	93·6	14·2	94·2	18·1	95·5	9·6
More than normal	0	2·1	2·8	11·1	2·0	4·8	1·6	5·7
Energetic								
Less than normal	5·1	12·1	6·5	11·9	5·0	15·2	4·8	10·3
Normal	90·3	15·8	90·4	14·8	91·4	16·1	92·4	12·7
More than normal	5·1	7·6	3·13	8·74	4·6	6·0	3·9	7·6
Stressed								
Less than normal	3·2	8·2	4·01	13·1	0·9	2·8	1·4	4·1
Normal	89·1	19·2	90·10	18·3	96·1	15·4	94·6	14·7
More than normal	7·5	5·4	5·89	12·1	2·7	4·6	4·0	13·0

* Mean value was significantly different from that for maltodextrin (*P* < 0·05).

† Mean value was significantly different from that at baseline (*P* < 0·05).

‡ Percentage coverage of each category over the total number of responses per volunteer was determined.

### SCFA analysis

Faecal SCFA concentrations were similar following Predilife and maltodextrin interventions (Table 4).

**Table 4.** SCFA profiles (mmol/g faeces) determined by GC from the placebo-controlled, double-blind, cross-over human feeding study with thirty-eight healthy human volunteers investigating the effects of Predilife (5 g/d) as compared with the placebo maltodextrin (5 g/d) (Mean values and standard deviations)

	Predilife				Maltodextrin			
	Baseline		Treatment		Baseline		Treatment	
SCFA	Mean	sd	Mean	sd	Mean	sd	Mean	sd
Acetate	0·7	0·4	0·8	0·7	0·6	0·3	0·7	0·4
Propionate	0·3	0·2	0·3	0·3	0·3	0·2	0·3	0·3
Butyrate	0·2	0·1	0·2	0·2	0·2	0·1	0·2	0·5
Total	1·2		1·3		1·1		1·2	

### Faecal secretory IgA and PGE_2_ levels

There were no significant differences in faecal sIgA concentrations or PGE_2_ levels when consuming Predilife or placebo (Table 5).

**Table 5.** Changes in faecal secretory IgA (sIgA) and PGE_2_ levels in the placebo-controlled, double-blind, cross-over human feeding study with thirty-eight healthy human volunteers investigating the effects of Predilife (5 g/d) as compared with the placebo maltodextrin (5 g/d) (Mean values and standard deviations)

	Baseline		Predilife treatment		Maltodextrin treatment	
Immunological parameter	Mean	sd	Mean	sd	Mean	sd
Faecal sIgA (µg/g)	628·3	80·8	623·3	80·8	628·3	80·9
Faecal PGE_2_ (pg/g)	1124·9	143·7	984·8	132·1	1056·3	89·3

### Denaturing gradient gel electrophoresis

**Fig. 2.**
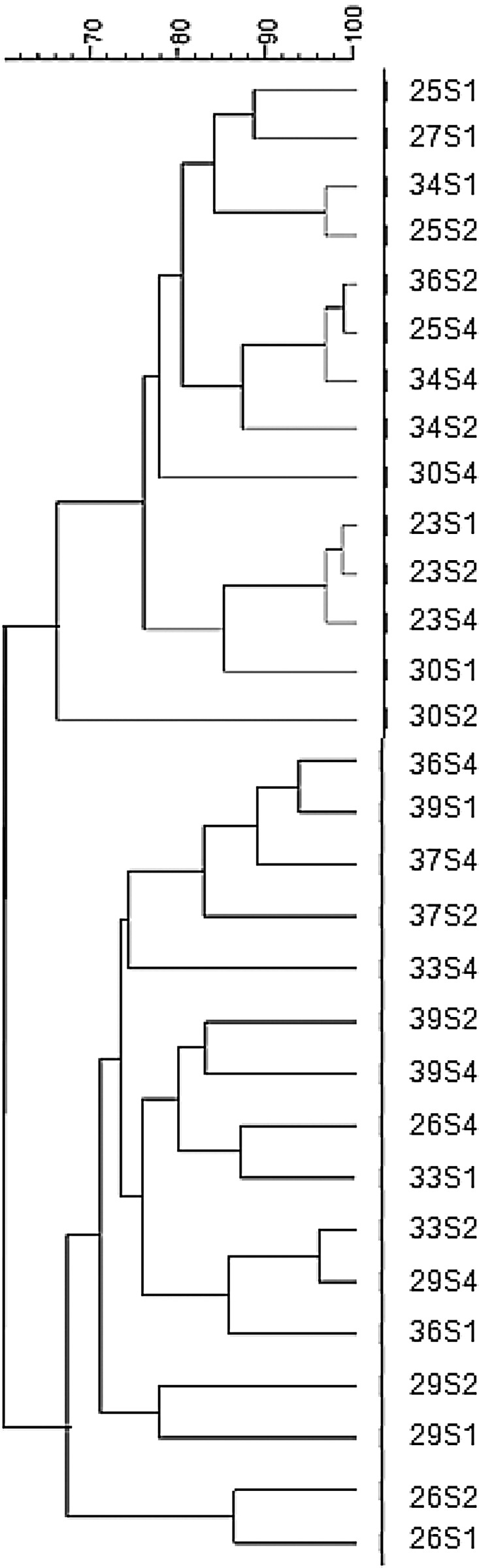
Cluster analysis: dendrogram of electrophoretic band pattern of subjects obtained using universal primers in faecal samples collected at baseline (S1), and during intake of Predilife (S2) and maltodextrin (S4).

DGGE results demonstrated that inter-individual variability was a greater variable than the typology of treatment. For group 1 with Predilife as the first treatment, nine of the nineteen subject samples were clustered along the intervention. For group 2 which received maltodextrin as the first treatment, only three of the nineteen subject samples were grouped along the intervention. The overall fingerprint of the gut microbiota did not differ significantly and most samples were randomly dispersed (Fig. 2).

### Microflora and SCFA changes in three-stage continuous culture models

To support the *in vivo* observations, we also investigated the effect of agave fructans on the growth of faecal bacteria in a three-stage continuous culture system. The changes in bifidobacterial and lactobacilli/enterococci populations measured by FISH in the three vessels at the two steady states (SS1 and SS2) are shown in Table 6. Significant increases in numbers of *Bifidobacterium* spp. and *Lactobacillus*/*Enterococcus* group were observed at SS2 in all three vessels after feeding with Synergy 1 and Predilife. No changes in bacterial numbers were observed with maltodextrin.

**Table 6.** Bacterial enumeration using fluorescent *in situ* hybridisation in the three-stage continuous system during two steady states for Predilife, Synergy 1 and maltodextrin† (Mean values and standard deviations)

			*Bifidobacterium* spp.		*Lactobacillus* spp.	
Substrate	Steady state	Vessel no.	Mean	sd	Mean	sd
Predilife	1	1	7·7	0·1	6·4	0·1
		2	7·6	0·1	6·5	0·1
		3	7·6	0·2	6·7	0·2
	2	1	8·3*	0·1	7·0*	0·1
		2	8·2*	0·1	6·9*	0·1
		3	7·8*	0·0	6·7*	0·0
Synergy 1	1	1	7·7	0·2	6·5	0·2
		2	7·8	0·1	6·7	0·1
		3	7·9	0·1	6·6	0·1
	2	1	8·2*	0·2	6·9*	0·2
		2	8·2*	0·1	6·8*	0·1
		3	8·1*	0·1	6·5*	0·1
Maltodextrin	1	1	7·7	0·2	6·7	0·2
		2	7·9	0·1	6·6	0·1
		3	7·9	0·1	6·6	0·1
	2	1	7·7	0·2	6·5	0·2
		2	7·8	0·1	6·6	0·4
		3	7·9	0·1	6·6	0·1

* Mean value was significantly from that during steady state 1 (*P* < 0·05).

† Bacterial numbers were measured as log_10_ cells/ml. Measurements were performed on three consecutive days during each steady state (after feeding the model with the respective substrate). Data from the 3 d were averaged.

The production of SCFA in the presence of Predilife, Synergy 1 and maltodextrin in the three-stage continuous culture models is shown in Table 7. Concentrations of acetate, propionate, butyrate and total fatty acids increased significantly in all three vessels upon Predilife and Synergy 1 dosing. However, SCFA in the presence of maltodextrin did not differ significantly. Also, no significant differences were observed for other SCFA produced.

**Table 7.** SCFA concentrations in the three-stage continuous system during two steady states for Predilife, Synergy 1 and maltodextrin† (Mean values and standard deviations)

			Acetic acid		Propionic acid		Butyric acid		Total SCFA	
Substrate	Steady state	Vessel no.	Mean	sd	Mean	sd	Mean	sd	Mean	sd
Predilife	1	1	0·6	0·2	1·2	0·1	0·0	0·1	1·8	0·1
		2	0·9	0·1	0·5	0·0	0·1	0·0	1·5	0·0
		3	0·3	0·1	0·1	0·00	0·1	0·0	0·4	0·0
	2	1	31·8*	0·1	10·2*	0·0	7·1*	0·0	49·1*	0·3
		2	27·0*	0·0	8·2*	0·0	5·2*	0·0	40·4*	0·0
		3	12·5*	0·0	12·5*	0·0	4·2*	0·0	28·2*	0·1
Synergy 1	1	1	1·1	0·1	0·5	0·1	0·2	0·2	1·8	0·1
		2	1·6	0·1	0·2	0·1	0·1	0·1	2·0	0·1
		3	0·8	0·2	1·3	0·2	0·1	0·8	3·2	0·8
	2	1	28·5*	0·1	12·8*	0·1	5·9*	0·1	47·9*	0·1
		2	15·2*	0·1	13·6*	0·1	4·6*	0·1	33·6*	0·1
		3	10·0*	0·0	5·9*	0·2	2·8*	0·0	18·9*	0·0
Maltodextrin	1	1	2·0	0·0	2·2	0·0	1·2	0·0	11·4	0·0
		2	1·2	0·0	1·0	0·0	1·5	0·0	5·6	0·0
		3	1·7	0·0	1·5	0·0	1·0	0·1	2·9	0·0
	2	1	2·1	0·0	1·0	0·0	0·04	0·1	11·2	0·0
		2	1·1	0·0	1·0	0·0	1·0	0·0	5·5	0·0
		3	1·2	0·0	1·1	0·0	1·0	0·0	3·2	0·0

* Mean value was significantly from that during steady state 1 (*P* < 0·05).

† SCFA concentrations were measured as mm. Measurements were performed on three consecutive days during each steady state (after feeding the model with the respective substrate). Data from the 3 d were averaged.

## Discussion

This is the first randomised, cross-over, double-blind clinical trial that has examined the effect of branched agave fructan on bacterial populations, SCFA, sIgA, PGE_2_, bowel habits and mood changes in healthy human volunteers.

Agave fructans increased bifidobacteria by 0·4 log in comparison with placebo (Table 2). The increase was similar to that observed with other intervention studies which report increases between 0·5 and 1·0 log bifidobacterial counts^(^^3^^,^^32–^^34^^)^. In a few reports where higher bifidobacterial numbers have been reported, higher doses of fructans were consumed^(^^35^^,^^36^^)^. In addition, the magnitude of change in bifidobacterial numbers also depends on initial levels^(^^30^^,^^32–^^34^^)^. Bifidobacterial numbers returned to baseline levels at the end of washout similar to previous studies^(^^33^^,^^34^^,^^36^^–^^38^^)^.

In addition to the increase in bifidobacterial counts, there was an increase in the lactobacilli/enterococci group: 0·4 log_10_ cells/g faeces compared with placebo (Table 2). This is similar to previous reports with fructans from chicory^(^^3^^)^ and globe artichoke^(^^32^^)^ where increases in lactobacilli/enterococci have been observed. There have been a few reports with fructans from Jerusalem artichoke^(^^33^^,^^35^^)^ or chicory^(^^34^^)^ where either no or less change in the lactobacilli/enterococci group has been reported.

There was no change in the numbers of total bacteria, *Atopobium*, *Bacteroides*, *Eubacterium rectale*/*Clostridium coccoides*, *E. coli* and *Roseburia*/*Eubacteria* group. This contrasts with decreases in levels of *Bacteroides* and clostridia reported in other studies with globe artichoke and Jerusalem artichoke inulin^(^^32^^,^^35^^)^. Overall, there were no significant differences in faecal SCFA concentrations. It is recognised that over 95 % of SCFA produced in the human large intestine is absorbed, with only a small proportion is excreted in faeces^(^^31^^–^^33^^,^^39^^)^.

Parallel to the human trial, the dynamics of bacterial growth and fermentation induced in the presence of Predilife was assessed and compared with Synergy 1 and maltodextrin using a three-stage continuous culture models. Both agave- and chicory-derived inulin stimulated the growth of bifidobacteria and lactobacilli to a similar extent (0·4–0·6 log_10_ cells/ml) as with the human trial data. However, the SCFA data indicated an increased production of acetate, propionate and butyrate, which contrasted with the human study data. This presumably reflected the fact that SCFA were rapidly absorbed by the human colon^(^^35^^)^. It may also suggest that the fructans were rapidly fermented in the proximal colon and thus not excreted in the faeces^(^^40^^)^. Further, the bacterial groups that were active agave degraders do not produce butyrate. This can be explained by substantial information on cross-feeding of fermentation products by gut microbiota^(^^41^^,^^42^^)^. The results from the *in vivo* and *in vitro* trials with branched agave fructans were consistent with those for linear fructans^(^^3^^,^^32–^^34^^)^. This indicates that structural differences between linear inulin type (β2-1 linkages) and branched agave (β2-1; 2-6 linkages) fructans do not seem to influence modulation of gut microbiota or their fermentation profiles.

As the extent of fermentation is also influenced by the degree of polymerisation, it may be suggested that the lower chain lengths in Predilife (degree of polymerisation 3–30)^(^^15^^)^ may contribute to the prebiotic effect. Further investigation of the fractions of agave fructans to identify chain lengths responsible for these effects may be warranted.

The effect of Predilife consumption on bowel habits and quality of life of subjects was assessed during the trial. Predilife did not influence the measured aspects of quality of life: mood, alertness, energy and stress levels. However, effects on bowel habits were more profound. No significant changes in stool frequency or consistencies were observed. There was no significant increase in abdominal pain levels. However, significantly increased intestinal bloating (mild to moderate) and flatulence (mild and severe) were recorded by subjects when consuming Predilife. Several studies have reported stimulation of bowel movements, and increased bloating and flatulence on ingestion of fructans^(^^32^^,^^33^^,^^35^^,^^36^^,^^39^^,^^43^^,^^44^^)^. The formation of H_2_, which is a metabolic endproduct of bacterial fermentation in the colon, is a major cause of flatulence. However, it must be noted that bifidobacteria, the numbers of which were significantly increased on intake of Predilife, are not considered to be producers of H_2_, or any other gas. In contrast, clostridia are prolific gas producers, but did not show any significant increase upon ingestion of Predilife. Thus, the relationship between specific intestinal bacteria and gas production remains to be clarified^(^^35^^,^^45^^)^.

The effect of Predilife consumption on the faecal immune markers sIgA and PGE_2_ was also determined. No change in these markers was observed. Faecal sIgA is primarily involved in mucosal immunity and protein barrier function against infection^(^^46^^)^. PGE_2_ plays a role in immune modulation and normal physiological gastrointestinal functions including cytoprotection^(^^47^^)^. In previous studies with pre- and probiotics, increases in immune markers^(^^46^^,^^47^^)^, unchanged levels^(^^19^^,^^48^^,^^49^^)^ and decreased levels have been reported^(^^19^^,^^50^^)^. Many factors including stress, exercise and dietary fat may have an impact on these immune parameters^(^^19^^,^^51^^,^^52^^)^, therefore resulting in variable trends. DGGE analyses, which allow a semi-qualitative evaluation of the fingerprint of the gut microbiota of subjects enrolled in the study, indicated that the treatment did not significantly modify the overall fingerprint of the gut microbiota to a great extent (to overcome the inter-individual differences). No significant differences were indicated between Predilife and maltodextrin.

In conclusion, *in vivo* and *in vitro* data confirm the prebiotic effectiveness of agave fructans as observed by selective increases in bifidobacteria and lactobacilli populations. Agave represents an important alternative prebiotic to other sources of fructans. In addition, due to its good solubility in cold water, it can be readily incorporated into beverages, dairy products, cheese and yogurts.
